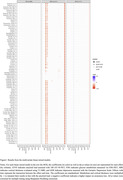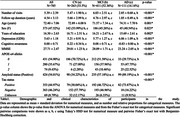# Exploring Neural Correlates of Cognitive Awareness across the Alzheimer’s Disease Continuum: A Multimodal Study

**DOI:** 10.1002/alz.089327

**Published:** 2025-01-03

**Authors:** Federica Cacciamani, Marion Houot, Elina Thibeau‐Sutre, Sophie Tezenas du Montcel, Raffaella Migliaccio

**Affiliations:** ^1^ Paris Brain Institute, Sorbonne, CNRS, Inserm, Paris France; ^2^ Qairnel, Paris France; ^3^ Sorbonne Université, Paris Brain Institute (ICM), INSERM, CNRS, CENIR MEG‐EEG, Paris France; ^4^ Assistance Publique Hopitaux de Paris (APHP), Paris France; ^5^ Institute of Memory and Alzheimer’s Disease, Salpêtrière University Hospital, Paris France; ^6^ University of Twente, Mathematics of Imaging and AI, Enschede Netherlands; ^7^ APHP, Paris France; ^8^ Sorbonne Université, Paris Brain Institute – Institut du Cerveau – ICM, Inserm U1127, CNRS UMR 7225, AP‐HP ‐ Hôpital Pitié‐Salpêtrière, Paris France; ^9^ AP‐HP, Groupe Hospitalier Pitié‐Salpêtrière, Department of Neurology, IM2A, Paris France

## Abstract

**Background:**

Anosognosia, a hallmark of Alzheimer’s disease (AD), manifests as a gradual decline in disease awareness, yet its neural correlates remain unclear. This study investigates how amyloid accumulation, glucose hypometabolism and cortical atrophy relate to cognitive awareness across the AD continuum. Both the pathological processes and the brain regions involved were studied.

**Methods:**

We included 263 cognitively‐normal (CN) individuals, 411 with mild cognitive impairment (MCI), and 111 diagnosed with AD from the ADNI cohort, with a mean±SD of 5.4±2.4 visits (Table 1). Awareness was assessed using the subject‐informant discrepancy on the ECog questionnaire, ranging from –3 to 3, with lower values indicating lower awareness. Neuroimaging measures included amyloid load assessed with ^18^F‐AV‐45‐PET, glucose metabolism with FDG‐PET, and cortical thickness with T1‐MRI, across the entire brain segmented into 86 regions of interest (ROIs) using FreeSurfer. In multivariate linear mixed models, we modeled the awareness index with baseline amyloid load, metabolism, and cortical thickness in each ROI. Models were adjusted for sex, age, education, and depression. We also factored in interactions between these variables and time, while correcting for multiple comparisons.

**Results:**

Results are presented in Figure 1. In individuals with AD: awareness was lower at baseline than in the other groups (p<0.001); it decreased over time (β±SE = ‐0.21±0.04; p<0.001); and lower awareness was associated with lower education (β±SE = 0.07±0.02, p = 0.004) and reduced left posterior cingulate metabolism (standardized β±SE = ‐0.24±0.07, p = 0.042). In participants with MCI: awareness was lower at baseline than in CN participants and higher than in AD (all p<0.001), and it decreased over time (β±SE = ‐0.07±0.01, p<0.001); greater decline in awareness was associated, first, with higher baseline amyloid in all ROIs except the hippocampus, parahippocampus, right posterior cingulate and right amygdala (all p<0.001); second, with decreased left posterior cingulate metabolism (standardized β±SE = ‐0.14±0.04, p = 0.006); third, with greater atrophy at baseline in the bilateral hippocampi, amygdala, and superior occipital gyri (all p<0.001). In CN individuals, we observed no significant associations (all p>0.05).

**Conclusion:**

All three processes were associated with cognitive awareness, exhibiting distinct patterns across different groups. Additionally, our findings revealed widespread involvement of multiple ROIs.